# Biocompatibility and inflammatory response of silver tungstate, silver molybdate, and silver vanadate microcrystals

**DOI:** 10.3389/fbioe.2023.1215438

**Published:** 2023-07-20

**Authors:** Bruna Natália Alves da Silva Pimentel, Sarah Raquel De Annunzio, Marcelo Assis, Paula Aboud Barbugli, Elson Longo, Carlos Eduardo Vergani

**Affiliations:** ^1^ School of Dentistry, São Paulo State University (Unesp), Araraquara, Brazil; ^2^ Departament of Physical and Analytical Chemistry, University Jaume I (UJI), Castelló, Spain; ^3^ CDMF, Federal University of São Carlos (UFSCar), São Carlos, Brazil

**Keywords:** silver-based metal oxides, cytokines, matrix metalloproteinases, reactive oxygen species, monocytes, macrophages

## Abstract

Silver tungstate (α-Ag_2_WO_4_), silver molybdate (β-Ag_2_MoO_4_), and silver vanadate (α-AgVO_3_) microcrystals have shown interesting antimicrobial properties. However, their biocompatibility is not yet fully understood. Cytotoxicity and the inflammatory response of silver-containing microcrystals were analyzed in THP-1 and THP-1 differentiated as macrophage-like cells, with the alamarBlue™ assay, flow cytometry, confocal microscopy, and ELISA. The present investigation also evaluated redox signaling and the production of cytokines (TNFα, IL-1β, IL-6, and IL-8) and matrix metalloproteinases (MMP-8 and -9). The results showed that α-AgVO_3_ (3.9 μg/mL) did not affect cell viability (*p* > 0.05). α-Ag_2_WO_4_ (7.81 μg/mL), β-Ag_2_MoO_4_ (15.62 μg/mL), and α-AgVO_3_ (15.62 μg/mL) slightly decreased cell viability (*p* ≤ 0.003). All silver-containing microcrystals induced the production of O_2_^−^ and this effect was mitigated by Reactive Oxygen Species (ROS) scavenger and N-acetylcysteine (NAC). TNFα, IL-6 and IL-1β were not detected in THP-1 cells, while their production was either lower (*p* ≤ 0.0321) or similar to the control group (*p* ≥ 0.1048) for macrophage-like cells. The production of IL-8 by both cellular phenotypes was similar to the control group (*p* ≥ 0.3570). The release of MMP-8 was not detected in any condition in THP-1 cells. Although MMP-9 was released by THP-1 cells exposed to α-AgVO_3_ (3.9 μg/mL), no significant difference was found with control (*p* = 0.7). Regarding macrophage-like cells, the release of MMP-8 and -9 decreased in the presence of all microcrystals (*p* ≤ 0.010). Overall, the present work shows a promising biocompatibility profile of, α-Ag_2_WO_4_, β-Ag_2_MoO_4_, and α-AgVO_3_ microcrystals.

## 1 Introduction

In recent years, several studies have evaluated the antimicrobial properties of medical materials functionalized with nanoparticles or antibiotics to improve their properties and prevent infections (Tran and Webster, 2013; Castro et al., 2014; Zhu et al., 2014; Castro et al., 2016a; Castro et al., 2016b; Hogan et al., 2019; Rangel et al., 2020; Verza et al., 2021). Silver has been used for centuries to treat infections and the use of silver and silver-containing particles has increased in the past few years (Politano et al., 2013). The literature shows that this metal has antimicrobial properties against a variety of microorganisms, such as *Staphylococcus aureus*, *Escherichia coli*, *Pseudomonas aeruginosa* and *Candida albicans* (Kim et al., 2009; Panáček et al., 2009; Martínez-Gutierrez et al., 2012; Dizaj et al., 2014; Shang et al., 2019). However, a limited number of studies have investigated its biocompatibility (Zhu et al., 2014; Rangel et al., 2020; Verza et al., 2021).

According to the literature, depending on the size and concentration of the particle, silver can decrease cell metabolism, increase ROS production, cytokine release and even induce programmed cell death (Foldbjerg et al., 2009; Liu et al., 2010; Foldbjerg et al., 2011; Park et al., 2011; Martínez-Gutierrez et al., 2012; Murphy et al., 2016). To optimize the antimicrobial properties and improve the biocompatibility of silver, some investigators have combined this metal with different metal oxides, such as vanadate (VO_3_), tungstate (WO_4_) and molybdate (MoO_4_) (Fabbro et al., 2016; Foggi et al., 2017a; Foggi et al., 2017b; Pimentel et al., 2020; Pimentel et al., 2022). Previous studies have shown that silver tungstate (α-Ag_2_WO_4_), silver molybdate (β-Ag_2_MoO_4_) and silver vanadate (α-AgVO_3_) showed no cytotoxic effect on normal oral keratinocytes (NOK-si) and gingival fibroblasts (FGH) (Haro Chávez et al., 2018; Assis et al., 2019; Pimentel et al., 2020). Furthermore, studies have shown that α-Ag_2_WO_4_ and α-AgVO_3_ do not promote DNA degradation (Haro Chávez et al., 2018; Pimentel et al., 2020). However, despite these promising findings, the biocompatibility of these materials could not be guaranteed until specific studies addressing oxidative stress, inflammatory responses, and extracellular matrix pathways were conducted.

The inflammatory response is a complex, multi-step process that occurs during injury and infection (Turner et al., 2014; Tu et al., 2022). Inflammation is part of the immune response and aims to eliminate the offending agent and initiate the healing process leading to tissue and functional restoration (Tu et al., 2022). The literature reports that silver particles, especially nanoparticles, have unique chemical and physical properties responsible for their antimicrobial activity. It is already known that metallic particles can indirectly induce the production of ROS due to the presence of metallic ions (Haro Chávez et al., 2018; Assis et al., 2019). According to the literature, ROS, including the superoxide anion (O2• -), activates the NF-κB (nuclear factor kappa B) and MAPK (mitogen-activated protein kinase) pathways, which are responsible for stimulating IL-1β, TNFα and IL—6 genes (Ndengele et al., 2005; Martínez-Gutierrez et al., 2012; Murphy et al., 2016; Yu et al., 2020; Canaparo et al., 2021). Thus, the presence of ROS can activate the immune response (Akter et al., 2018) and stimulate the immune system to produce various cytokines and other inflammatory mediators (Parks et al., 2004; Abdulkhaleq et al., 2018).

Given the potential application of α-Ag_2_WO_4_, β-Ag_2_MoO_4_ and α-AgVO_3_ in dental materials and medical devices to prevent oral infections, it is imperative to establish their ability to mitigate any excessive inflammatory responses. Furthermore, the role of matrix metalloproteinases (MMPs), which are responsible for tissue remodeling and healing (Araújo et al., 2011), must be understood. Previous studies have shown that MMPs are strongly associated with periodontal disease, leading to the loss of periodontal attachment and bone destruction (Franco et al., 2017; Al-Majid et al., 2018). Among the 23 types of MMPs already identified, the upregulation of MMP-8 and -9 has been related to periodontitis and peri-implantitis (Franco et al., 2017; Checchi et al., 2020) and it is associated with disease progression and bone loss (Arakawa et al., 2012; Al-Majid et al., 2018). High levels of MMP-8 and -9 are found in periodontal tissues where the disease is established, possibly indicating severity and progression of the pathology (Franco et al., 2017; Al-Majid et al., 2018; Checchi et al., 2020). Additionally, MMP production can contribute to the failure of dental restorations (Hashimoto et al., 2016). Therefore, therapies aimed at controlling MMP production, while avoiding cytotoxic and genotoxic effects and reducing their levels, have the potential to effectively prevent periodontal disease and peri-implantitis.

In this context, the present study evaluated the cytotoxicity profile, and the production of reactive oxygen species (ROS), pro-inflammatory cytokines (IL-1β, TNFα, IL-6, and IL-8), and MMPs (−8 and −9), by THP-1 cells (human monocytes) and THP-1 macrophage-like cells following exposure to silver-containing microcrystals (α-Ag_2_WO_4_, β-Ag_2_MoO_4_ and α-AgVO_3_).

## 2 Materials and methods

### 2.1 Preparation of microcrystals

Silver tungstate, silver molybdate, and silver vanadate were prepared as previously described (Fabbro et al., 2016; Foggi et al., 2017a; Oliveira et al., 2017). Briefly, 1 × 10^−3^ mol of silver nitrate (AgNO_3_; 99.98% purity; Cennabras, Guarulhos, SP, Brazil) was diluted in 50 mL of distilled water. Simultaneously, 5 × 10^−4^ mol of sodium tungstate dihydrate (Na_2_WO_4_•2H_2_O; 99.99% purity; Sigma-Aldrich, St. Louis, MO, United States) or sodium molybdate dihydrate (Na_2_MoO_4_•2H_2_O; 99.98% purity; Alfa Aesar, Haverhill, MA, United States) or 1 × 10^−3^ mol of ammonium metavanadate (NH_4_VO_3_; 99.99% purity; Sigma-Aldrich, St. Louis, MI, United States) were diluted in 50 mL of distilled water. Temperatures of 70°C for α-Ag_2_WO_4_ and β-Ag_2_MoO_4_ and 10°C for α-AgVO_3_ were used. After reaching the temperatures required, the solutions were mixed, instantly forming a precipitate. These precipitates were washed with distilled water to a pH of 7 and oven-dried at 60°C for 12 h. After synthesis, all microcrystals were diluted in PBS to 2 mg/mL (stock solution), and the samples were maintained in the dark and at room temperature until further use.

### 2.2 Physicochemical assessment and silver concentration

The structural characterization of the materials was performed at long-range, a D/Max-2500 PC diffractometer (Rigaku, Japan) with Cu Kα radiation (*λ* = 1.54056 Å) in the 2θ range of 10°–80° at a scan rate of 0.01°min^−1^. To analyze the morphologies, a scanning electron microscope with a field emission gun (FEG-SEM) FEI Model Inspect F50, operating at 5 kV was used. Particle count analysis was performed using ImageJ software, with a minimum count of 100 particles. The silver content present in the microcrystals suffers oxidation during the synthesis process. To calculate the amount of oxidized silver [Ag^+^] in the microcrystals structure, first, the microcrystal concentration was converted from µg/mL to µmol/mL using the following equation: silver content in each microcrystal concentration = microcrystal concentration (µmol/mL) × 10^–6^/Molecular Weight of the microcrystal. Then, the amount of silver was calculated based on the number of mols released by each microcrystal.

### 2.3 Microcrystals concentration against *Candida albicans*

The experimental groups were defined based on the minimal inhibitory concentration (MIC) and minimal fungicidal concentration (MFC) from *C. albicans* ATCC 90028 performed previously by Fabbro et al. (2016); Foggi et al. (2017a),; Pimentel et al. (2020). Both α-Ag_2_WO_4_ and β-Ag_2_MoO_4_ presented the same MIC and MFC values (7.81 μg/mL and 15.62 μg/mL, respectively for each microcrystal). For α-AgVO_3_, the MIC and MFC values were 3.9 μg/mL and 15.62 μg/mL, respectively. Working solutions were prepared immediately before use by diluting each microcrystal stock solution in Dulbecco’s modified Eagle’s medium (DMEM).

### 2.4 *In vitro* THP-1 and macrophages-like cell culture and growth conditions

The cell line THP-1 (human monocytes from peripheral blood) was obtained from the Rio de Janeiro Cell Bank (BCRJ; cell line code 0234) and routinely cultured at 37°C in a 5% CO_2_-humidified environment in Roswell Park Memorial Institute medium (RPMI-1640; Sigma-Aldrich, St. Louis, MO, United States), supplemented with 2 mM of glutamine (LONZA, Basel, Switzerland), 10 mM of HEPES (Sigma-Aldrich, St. Louis, MO, United States), 1 mM of sodium pyruvate (Sigma-Aldrich, St. Louis, MO, United States), 4.5 g/L of glucose (Synth, Diadema, SP, Brazil), 1.5 g/L of sodium bicarbonate (Synth, Diadema, SP, Brazil), 1% of antibiotic/antimycotic solution (Sigma-Aldrich, St. Louis, MO, United States), 10% of fetal bovine serum (FBS; Gibco, Grand Island, NY, United States), and 0.09% of β-mercaptoethanol (Gibco, Grand Island, NY, United States). To obtain the macrophages-like from THP-1 cells, before each experiment, the THP-1 cells were seeded and stimulated with 100 ng/mL of phorbol 12-myristate 13-acetate (PMA; Sigma-Aldrich, St. Louis, MO, United States) (Park et al., 2007), which was added to the cell culture medium and maintained at 37°C in a 5% CO_2_-humidified environment to achieved the macrophage phenotype. After 48 h, the supernatant was discarded and the macrophages cells were washed twice with PBS. Subsequently, a fresh medium was added and maintained overnight before the assays.

### 2.5 Cell viability assay

Cell viability was performed after 24 h of contact with silver-containing microcrystals, and it was assessed by alamarBlue™ assay. THP-1 and macrophages-like cells (1 × 10^6^/well) were seeded on 12-well plates at a final volume of 3 mL of RPMI medium with 5% FBS and maintained at 37°C in 5% CO_2_. After 16 h, the cells were washed with PBS, and the cell culture medium without FBS was added with silver-containing microcrystals (α-Ag_2_WO_4_: 7.81 μg/mL; β-Ag_2_MoO_4_: 15.62 μg/mL; α-AgVO_3_: 3.9 μg/mL and 15.62 μg/mL). The plates were maintained at 37°C in 5% CO_2_, and after 4 h and 24 h an aliquot of 100 µL of the supernatants from each well were collected and stored at −20°C until the cytokine production assay. After 24 h of contact with the microcrystals, the cells were incubated for 4 h in a fresh cell culture medium containing 10% of alamarBlue™ reagent (Invitrogen, Carlsbad, CA, United States). Then, 200 µL of each well was transferred in quadruplicate to a black 96-well plate, and the fluorescence emission was measured (excitation: 544 nm; emission: 590 nm; Fluoroskan Ascent II, ThermoFisher Scientific, Waltham, MA, United States). Standard cell culture conditions were used as live cell control (CT) and cells incubated with 10 µL of lysis buffer solution (LB; Triton-x 100 9%; Sigma-Aldrich, St. Louis, MO, United States) were used as dead cell control. This assay was performed in quadruplicate and on three different occasions.

### 2.6 Intracellular ROS (O_2_^−^) quantification

The production of superoxide (O_2_^−^) induced by silver-containing microcrystals on THP-1 and macrophages-like cells was conducted with dihydroethidium reagent (DHE; D23107; Invitrogen, Carlsbad, CA, United States), a selectively probe for O_2_^−^detection. Cells were seeded in a 96-well plate at 2 × 10^4^ cells/well in Krebs-Henseleit buffer (pH 7.0 ± 0.2). Then, 200 µL of DHE (1:1000) was added to each well and the plates were maintained at 37°C for 1 h. Further, the α-Ag_2_WO_4_ (7.81 μg/mL), β-Ag_2_MoO_4_ (15.62 μg/mL), and α-AgVO_3_ (3.9 μg/mL and 15.62 μg/mL) silver-containing microcrystals were added to the corresponding wells and the plates were maintained at 37°C for 1 h. Thereafter, the cells were washed and, 100 µL of fresh Krebs-Henseleit buffer was added to each well. The intracellular superoxide production was measured by fluorescence emission in a fluorescence reader (FLUOstar Omega, BMG Labtech, Cary, NC, United States; Ex.: 540-10nm; Em.: 620–10 nm). Cells under standard culture conditions were used as negative O_2_^−^ control, hydrogen peroxide (H_2_O_2_ [0.125 mM]; Sigma-Aldrich, St, Louis, MO, United States) was used as positive O_2_^−^ control, and N-Acetyl-L-cysteine (NAC) [0.01 mM] (Sigma-Aldrich, St. Louis, MO, United States) as a scavenger. This assay was performed in quadruplicate and on three different occasions.

### 2.7 Intracellular ROS (O_2_^−^) detection by confocal laser scanning microscopy (CLSM)

For the CLSM assay, THP-1 and macrophage-like cells were seeded in a 48-well plate at 3 × 10^4^ cells/well and incubated with DHE probe for 1 h at 37°C in 5% CO_2_. Then, the α-Ag_2_WO_4_ (7.81 μg/mL), β-Ag_2_MoO_4_ (15.62 μg/mL), and α-AgVO_3_ (3.9 μg/mL and 15.62 μg/mL) microcrystals were added to the corresponding wells and the plate were incubated for another hour at 37°C in 5% CO_2_. The probe excess was removed and the CLSM images were obtained with an LSM 800 microscope (Carl Zeiss, Oberkochen, Germany) using a 561-nm laser, detection of brightfield and fluorescence spectra up to 700 nm, with ×20 objective. Cells under standard culture conditions were used as negative O_2_^−^ control, hydrogen peroxide (H_2_O_2_ [0.125 mM]; Sigma-Aldrich, St. Louis, MO, United States) as positive O_2_^−^ control, and NAC [0.01 mM] (Sigma-Aldrich, St. Louis, MO, United States) as scavenger control.

### 2.8 Production of pro-inflammatory cytokines

The IL-1β, TNFα, IL-6, and IL-8 cytokines production was assessed after THP-1 and macrophage-like cells were exposed to α-Ag_2_WO_4_ (7.81 μg/mL), β-Ag_2_MoO_4_ (15.62 μg/mL), and α-AgVO_3_ (3.9 μg/mL and 15.62 μg/mL) microcrystals, at 4 and 24 h of exposure. The samples were obtained as described in section 2.5 and maintained at—20°C until the analysis. The Human Inflammatory Cytokine Kit (Cat. No. 551811; BD Biosciences, San Jose, CA, United States) was used according to the manufacturer’s instructions. Briefly, while the samples thawed at room temperature, the lyophilized Human Inflammatory Cytokines Standards were reconstituted with 2 mL of Assay diluent, and then a serial dilution was performed from 1:2 until 1:256. The negative control (0 pg/mL) was prepared only with Assay Diluent. Next, a mix of capture beads was prepared and 50 µL was added in each tube (standard curve and samples). Then, 50 µL of standard cytokines or samples were added to each corresponding tube, and finally 50 µL of Human Inflammatory PE Detection Reagent were added to all tubes. After 3 h of dark room incubation, 1 mL of Wash Buffer was added to all tubes and centrifuged at 200 *g* for 5 min. The supernatants were carefully discarded, and the pellets were resuspended in 300 µL of Wash Buffer. The samples were analyzed using a BD FACSAria™ Fusion Flow Cytometer (BD Biosciences, San Jose, CA, United States), and all data obtained were evaluated with the FCAP Array software v3 (BD Biosciences, San Jose, CA, United States).

### 2.9 MMPs signaling

To evaluate the production of MMP-8 and -9, THP-1 and macrophage-like cells were seeded in 25-cm^2^ flasks at a concentration of 5 × 10^5^ cells/flask in RPMI culture medium containing 5% FBS and 5% CO_2_ at 37°C. After 16 h, the cells were washed with PBS, and fresh culture medium, without FBS, containing α-Ag_2_WO_4_ (7.81 μg/mL), β-Ag_2_MoO_4_ (15.62 μg/mL), and α-AgVO_3_ (3.9 μg/mL and 15.62 μg/mL) microcrystals were added to the correspondent treatment flask. The cells were maintained at 37°C in 5% CO_2_ for 24 h. Negative control cells (CT) were maintained under standard cell culture conditions, and the positive control of MMP production was assessed with cells incubated with 1 μg/mL of lipopolysaccharide from *Escherichia coli* (LPS; Sigma-Aldrich, St. Louis, MO, United States). Subsequently, the supernatants were collected and stored at −20°C until analysis. This assay was performed in duplicate on two independent occasions. Before the ELISA assay, the amount of total protein in each sample was measured with the Bradford protein assay (Bradford, 1976) (Sigma-Aldrich, St Louis, MO, United States) using bovine serum albumin (BSA; Sigma-Aldrich, St. Louis, MO, United States) as the standard. Spectrophotometric measurements were performed at 595 nm (EZ Read 400 Microplate Reader; BioChrom, Cambourne, CAM, United Kingdom). The MMPs (−8 and −9) production was detected with the MMP-8 Human ELISA Kit (ab100609, Abcam, Cambridge, CBE, United Kingdom) and MMP-9 SimpleStep ELISA Kit (ab246539; Abcam, Cambridge, CBE, United Kingdom), according to the manufacturer’s instructions. The OD was immediately read at 600 nm using a microplate reader (EZ Read 400 Microplate Reader; BioChrom, Cambourne, CAM, United Kingdom). The final data were normalized by the amount of protein per sample. This assay was performed in triplicate in a single occasion.

### 2.10 Statistical analysis

All data obtained were analyzed for normality (Shapiro-Wilk’s test) and homoscedasticity (Levene test). The statistical analysis of cell viability and O_2_^−^ production was performed with one-way ANOVA, followed by Tukey’s *post hoc* on the IBM SPSS Statistics software (version 23). For cytokine and MMP production, a 95% confidence interval (CI) was defined to compare the results among groups. A significance level of 5% was adopted.

## 3 Results

### 3.1 Microcrystals’ characterization and silver concentration

**FIGURE 1:**
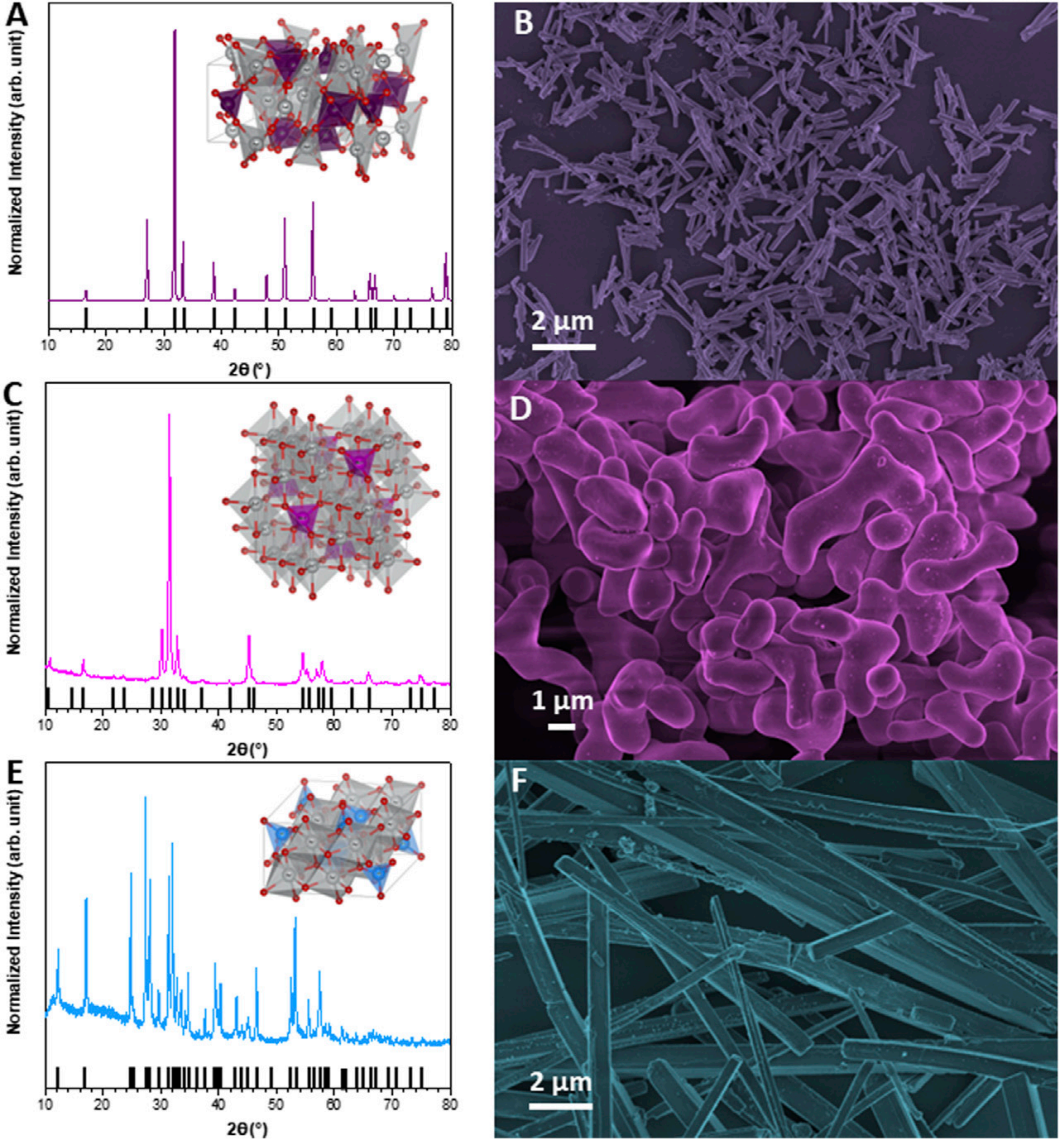
X-ray diffraction (XDR) patterns and FE-SEM images. **(A,B)** α-Ag2WO4; **(C,D)** β-Ag2MoO4; **(E,F)** α-AgVO3.

The XRD and FEG-SEM analyses are shown in Figure 1. For the α-Ag_2_WO_4_ sample, the orthorhombic phase was obtained, with a *Pn2n* space group (PDF 34–61) (Figure 1A). This phase has a complex structure, formed by several clusters of [AgO_x_] (x = 2, 4, 6, and 7) and distorted octahedral clusters of [WO_6_] (Assis et al., 2020). Its morphology is composed of hexagonal micro rods (Figure 1B) of average length and width of 0.95 ± 0.35 and 0.15 ± 0.06 µm, respectively. The β-Ag_2_MoO_4_ phase was also obtained, with cubic structure and *Fd-3m* space group (PDF 8–473) (Figure 1C). This structure has a lower complexity in terms of constituent clusters, being formed by distorted octahedral and tetrahedral clusters of [AgO_6_] and [MoO_4_], respectively (Foggi et al., 2020). Its morphology does not have a polyhedral microstructure, known as bean-like morphology (Figure 1D). These particles have a high degree of aggregation, coalescing in many cases. The average length and width obtained for this sample was 3.80 ± 0.80 and 1.40 ± 0.31 µm, respectively. For α-AgVO_3_, it is observed that the pure phase is obtained, without any additional peak, referring to the monoclinic phase with *C2/c* space group (PDF 89–4396) (Figure 1E). This phase is formed by distorted octahedral clusters of [AgO_6_] and distorted clusters of [VO_4_] (Silva et al., 2019). Its morphology is homogeneous, with the shape of 4-sided micro rods (Figure 1F). Its average length and width are 9.17 ± 4.98 and 0.52 ± 0.18 µm, respectively, showing high sample size dispersibility. The results obtained for the three samples are in agreement with those published in previous works (Oliveira et al., 2017; Assis et al., 2021; Teodoro et al., 2022).

To calculate the total of silver ions [Ag^+^] concentration found in the microcrystals, the target concentration of each one was converted from µg/mL to µmol/mL, as following:

(7.81 μg/mL)/(463.57 g/mol) of α-Ag_2_WO_4_ = 0.0168 μmol/mL of α-Ag_2_WO_4_;

(15.62 μg/mL)/(375.68 g/mol) of β-Ag_2_MoO_4_ = 0.0416 μmol/mL of β-Ag_2_MoO_4_;

(3.9 μg/mL)/(206.81 g/mol) of α-AgVO_3_ = 0.0188 μmol/mL of α-AgVO_3_;

(15.62 μg/mL)/(206.81 g/mol) of α-AgVO_3_ = 0.0755 μmol/mL of α-AgVO_3_.

Since, according to the chemical definition, 1 mol of α-Ag_2_WO_4_ and β-Ag_2_MoO_4_ releases 2 mol of Ag^+^ each, the molar concentration of Ag^+^ in these two microcrystals is twice the concentration of α-Ag_2_WO_4_ and β-Ag_2_MoO_4_, 0.0168 μmol/mL and 0.0416 μmol/mL, respectively. Thus, the total [Ag^+^] was 0.0336 μmol/mL or 0.0156 μg/mL for α-Ag_2_WO_4_ and 0.0832 μmol/mL or 0.0313 μg/mL for β-Ag_2_MoO_4_. In the same way, 1 mol of α-AgVO_3_ releases 1 mol of Ag^+^, so the total [Ag^+^] was 0.0188 μmol/mL or 0.0039 μg/mL for α-AgVO_3_ at 3.9 μg/mL, and 0.0755 μmol/mL or 0.0156 μg/mL for α-AgVO_3_ at 15.62 μg/mL.

### 3.2 Cell viability

**FIGURE 2:**
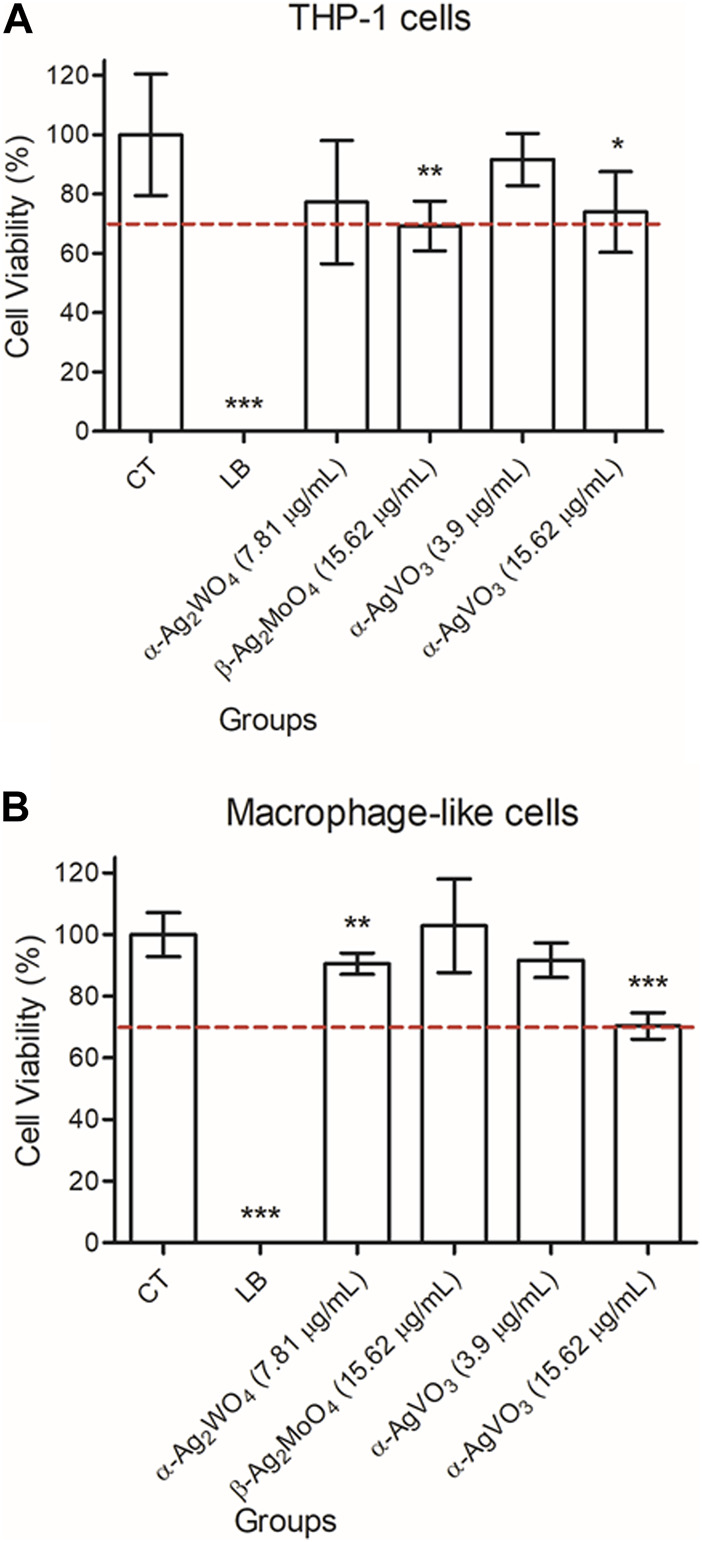
Mean values of cell viability (%) of **(A)** THP-1 cells and **(B)** macrophage-like cells after contact with α-Ag2WO4 (7.81 µg/mL), β-Ag2MoO4 (15.62 µg/mL), and α-AgVO3 (3.9 µg/mL and 15.62 µg/mL) microcrystals for 24 hours. Error bars: standard deviation. CT: live cell control; LB: lysis buffer, dead cell control. Dotted line: 70% of cell viability. Groups with asterisks are statistically different from control. *: p = 0.017; **: p < 0.009; ***: p < 0.0001. α= .05.

The cell viability was evaluated by alamarBlue™ assay (Figure 2). First, when THP-1 cells were maintained in contact with α-Ag_2_WO_4_ (7.81 μg/mL) and α-AgVO_3_ (3.9 μg/mL), cell viability was statistically similar to the control group (CT) (*p* > 0.05) (Figure 2A). However, the contact of THP-1 cells with β-Ag_2_MoO_4_ (15.62 μg/mL) and α-AgVO_3_ (15.62 μg/mL) promoted a decrease in cell viability as compared to CT (*p* = 0.0003 and *p* = 0.017, respectively) (Figure 2A). Similarly, the cell viability of macrophage-like cells was decreased after exposure to α-Ag_2_WO_4_ (7.81 μg/mL) and α-AgVO_3_ (15.62 μg/mL), when compared to CT (*p* = 0.0009 and *p* < 0.0001, respectively) (Figure 2B). The contact of α-AgVO_3_ (3.9 μg/mL) and β-Ag_2_MoO_4_ (15.62 μg/mL) microcrystals with macrophage-like cells promoted cell viability similar to CT (*p* = 0.05 and *p* = 0.991, respectively) (Figure 2B).

Despite the observed changes in cell viability, based on the cytotoxicity classification proposed by Lonnroth and Dahl (2001), Lönnroth and Dahl (2003) and Sletten and Dahl (1999), no cytotoxicity was noted when THP-1 cells were treated with α-AgVO_3_ (3.9 μg/mL; reduction of 8.31% in cell viability), whereas α-AgVO_3_ (15.62 μg/mL), α-Ag_2_WO_4_ (7.81 μg/mL), and β-Ag_2_MoO_4_ (15.62 μg/mL) were slightly cytotoxic (reduction of 26.04%, 22.7%, and 30.71% in cell viability, respectively). For macrophage-like cells, α-AgVO_3_ (3.9 μg/mL), α-Ag_2_WO_4_ (7.81 μg/mL), and β-Ag_2_MoO_4_ (15.62 μg/mL) were non-cytotoxic (reduction of 8.27%, 9.42%, and −2.84% in cell viability, respectively), whereas α-AgVO_3_ (15.62 μg/mL) presented slight cytotoxicity (reduction of 29.64% in cell viability).

### 3.3 Intracellular O_2_^−^ quantification and imaging

**FIGURE 3:**
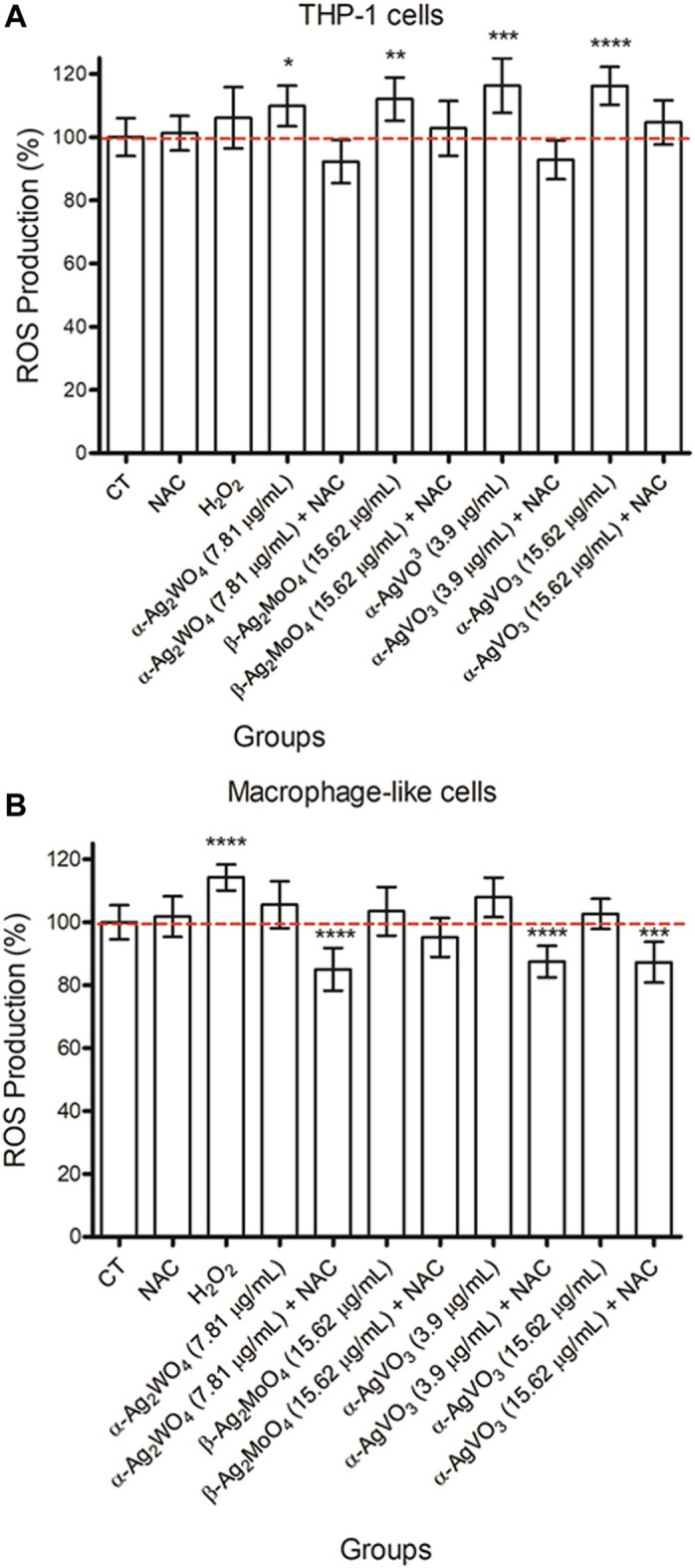
Mean values of fluorescence intensity of THP-1 cells **(A)** and macrophage-like cells **(B)** using DHE probe after contact with NAC [0.01 mM], H2O2 [0.125 mM], and α-Ag2WO4 (7.81 µg/mL), β Ag2MoO4 (15.62 µg/mL), and α-AgVO3 (3.9 µg/mL and 15.62 µg/mL) microcrystals (alone or with NAC). Error bars: standard deviation. NAC: N-Acetyl-L-cysteine; H2O2: hydrogen peroxide. Groups with asterisks are statistically different from control *: p = 0.026; **: p = 0.005; ***: p = 0.001; ****: p < 0.0001. α= .05.

The generation of O_2_^−^ by the cells after the contact with silver-containing microcrystals was evaluated with fluorescence emission using a DHE probe. The THP-1 cells exposed to silver-containing microcrystals increased the production of O_2_^−^. As expected, when cells were incubated with microcrystals and the NAC scavenger was added (+NAC), there was a drop in the O_2_^−^ production (Figure 3A). The highest decrease regarding O_2_^−^ production was observed when THP-1 cells were exposed to α-Ag_2_WO_4_ (7.81 μg/mL) + NAC and α-AgVO_3_ (3.9 μg/mL) + NAC, but yet they were similar to control group (CT; *p* > 0.170) (Figure 3A).

Macrophage-like cells showed lower production of O_2_^−^ compared to THP-1 cells, which was similar to control group (*p* > 0.05) (Figure 3B). Nevertheless, similar to THP-1, there was a decrease in the O_2_^−^ production in the presence of NAC when macrophage-like cells were exposed to all microcrystals, particularly to α-Ag_2_WO_4_ (7.81 μg/mL; *p* < 0.0001), α-AgVO_3_ (3.9 μg/mL; *p* < 0.0001) and α-AgVO_3_ (15.62 μg/mL; *p* < 0.001) (Figure 3B).

**FIGURE 4:**
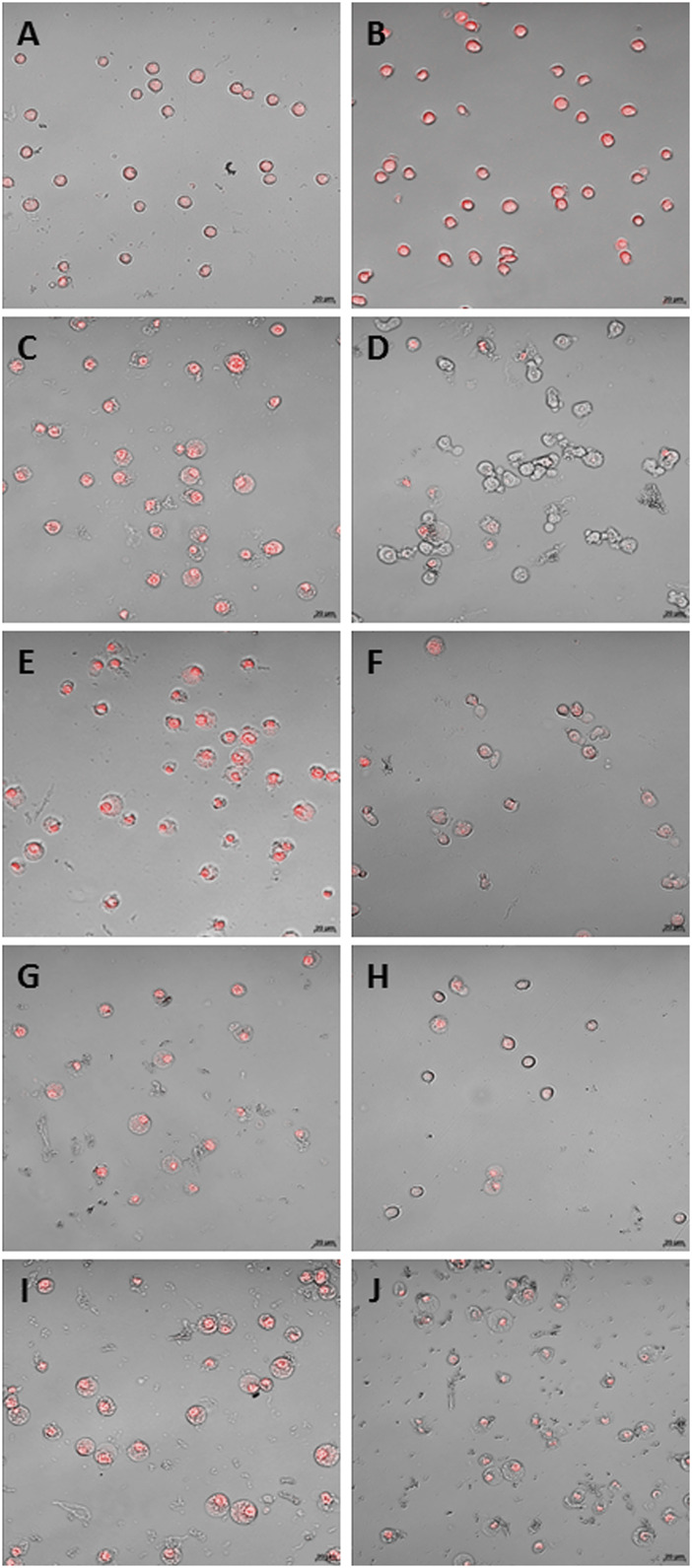
CLSM of THP-1 cells. **(A)** standard culture conditions; **(B)** H2O2 [0.125 mM]; C and D: α Ag2WO4 (7.81 µg/mL) without NAC **(C)** and with NAC [0.01 mM] **(D)**; E and F: β-Ag2MoO4 (15.62 µg/mL) without NAC **(E)** and with NAC [0.01 mM] **(F)**; G and H: α-AgVO3 (3.9 µg/mL) without NAC **(G)** and with NAC [0.01 mM] **(H)**; I and J: α-AgVO3 (15.62 µg/mL) without NAC **(I)** and with NAC [0.01 mM] **(J)**. Red fluorescence: O2—production.

**FIGURE 5:**
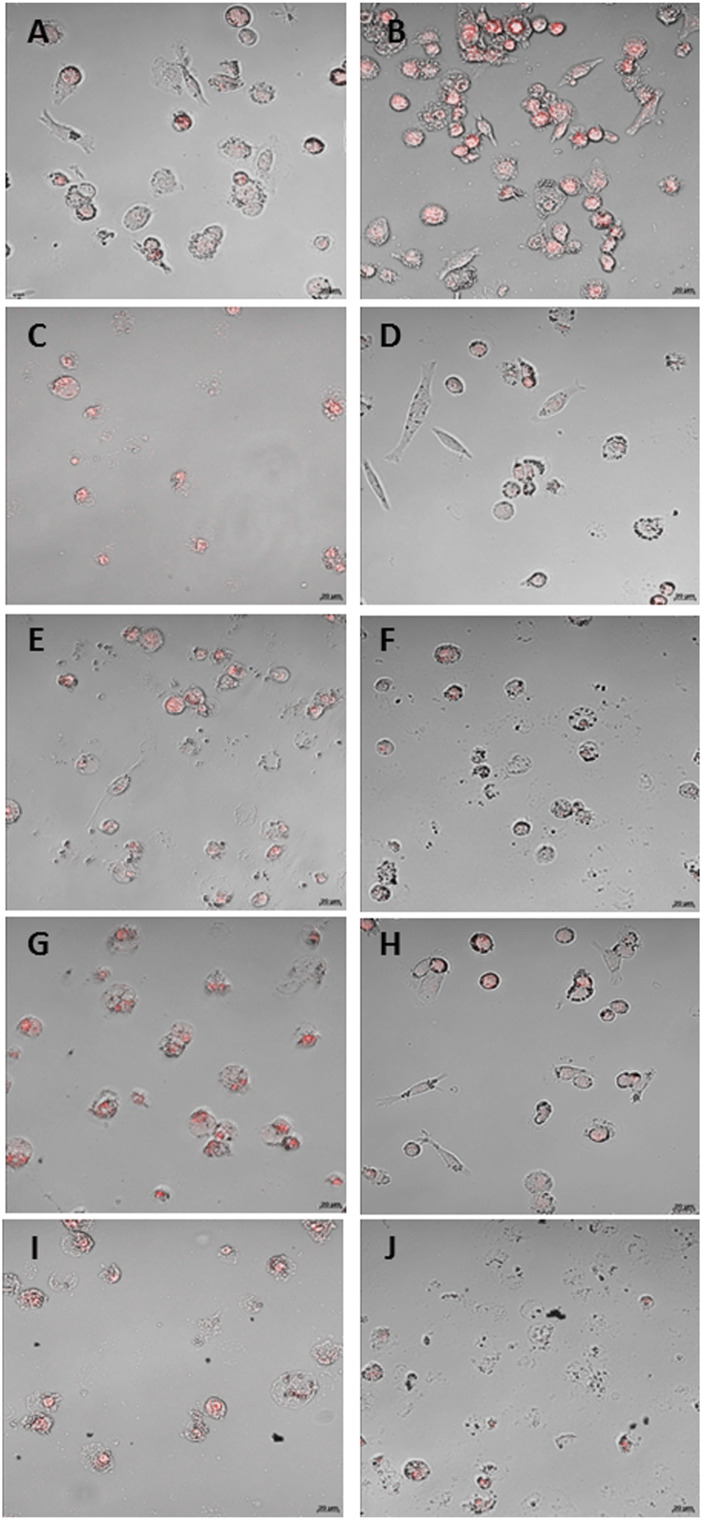
CLSM of macrophage-like cells. **(A)** standard culture conditions; **(B)** H2O2 [0.125 mM]; C and D: α-Ag2WO4 (7.81 µg/mL) without NAC **(C)** and with NAC [0.01 mM] **(D)**; E and F: β-Ag2MoO4 (15.62 µg/mL) without NAC **(E)** and with NAC [0.01 mM] **(F)**; G and H: α-AgVO3 (3.9 µg/mL) without NAC **(G)** and with NAC [0.01 mM] **(H)**; I and J: α-AgVO3 (15.62 µg/mL) without NAC **(I)** and with NAC [0.01 mM] **(J)**. Red fluorescence: O2—production.

The CLSM images confirmed the data obtained with the intracellular fluorescence emission quantification. The THP-1 (Figure 4) and macrophage-like cells (Figure 5) treated with H_2_O_2_ showed higher fluorescence than control (CT) (Figures 4A, B; Figures 5A, B, respectively). The treatment with microcrystals also presented high fluorescence, which was decreased when microcrystals were associated with NAC (Figures 4C–J; Figures 5C–J).

### 3.4 Production of pro-inflammatory cytokines

**FIGURE 6:**
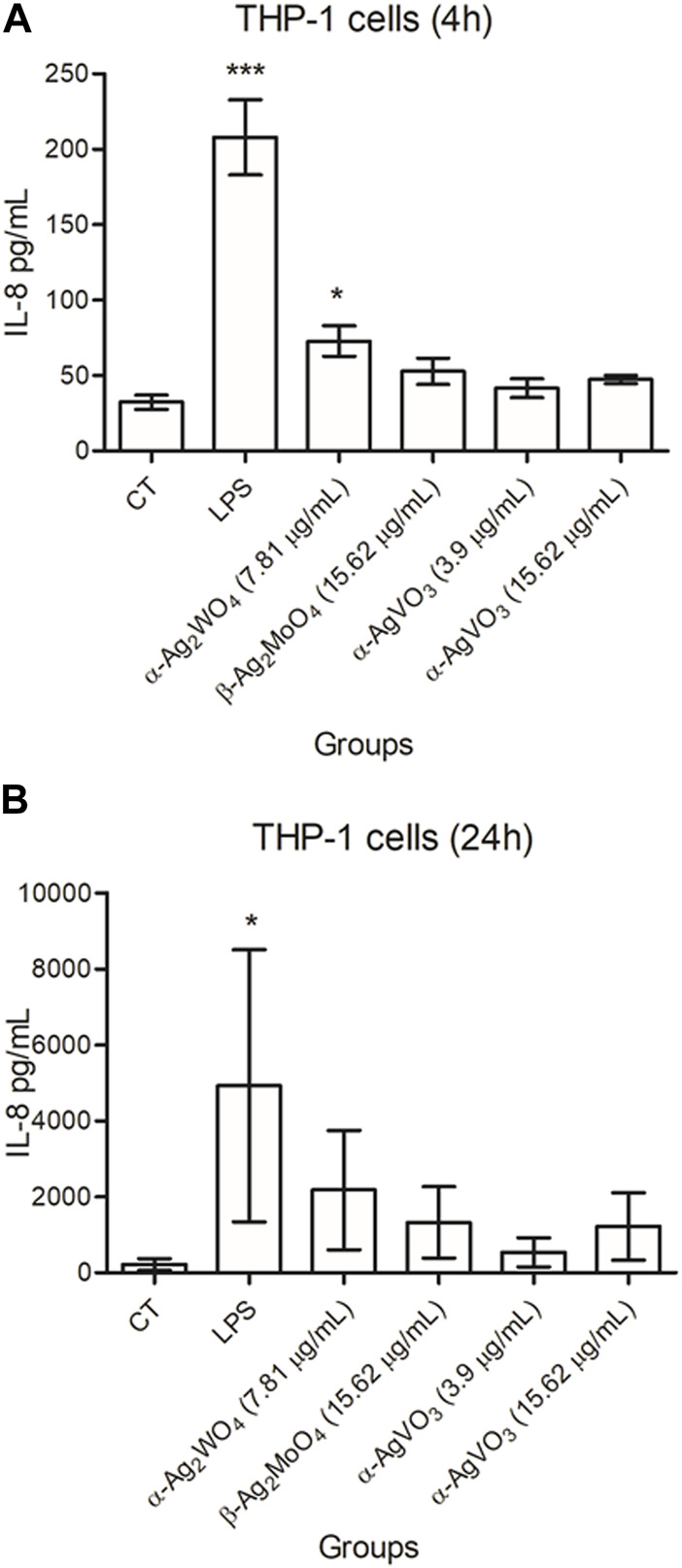
Mean values of pg/mL of IL-8 produced by THP-1 cells after 4 hours **(A)** and 24 hours **(B)** of contact with α-Ag2WO4 (7.81 µg/mL), β-Ag2MoO4 (15.62 µg/mL), and α-AgVO3 (3.9 µg/mL and 15.62 µg/mL) microcrystals. Error bars: standard deviation; CT: standard culture control; LPS: lipopolysaccharide, control. Groups with asterisks are statistically different from control. *: p < 0.0463; ***: p < 0.0001. α= .05.

The flow cytometry analysis showed that THP-1 cells produced only IL-8 at both 4 and 24 h (Figures 6A, B, respectively). For this cell line, only α-Ag_2_WO_4_ (7.81 μg/mL) was able to increase IL-8 production after 4 h of contact (*p* = 0.0136), when compared to the control group (CT). However, after 24 h of contact, there were no significant differences (*p* ≥ 0.7161) in the production of IL-8 between all experimental groups and the control group. The other cytokines evaluated (IL-1β, TNFα, and IL-6) were not detected in this cell line at any conditions (data not shown).

**FIGURE 7:**
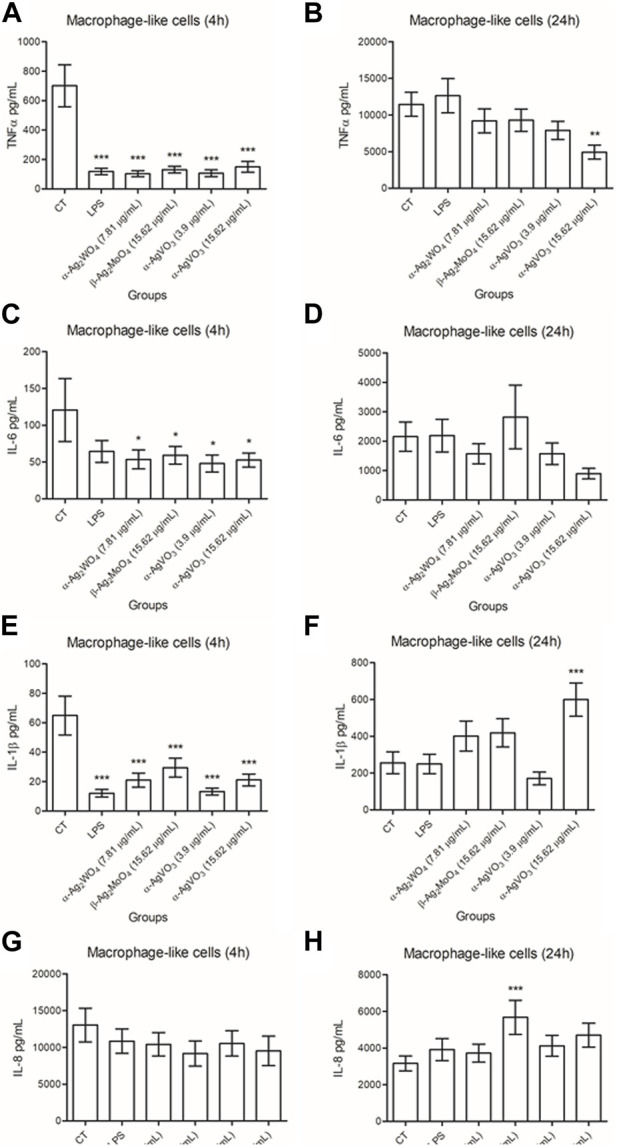
Mean values of pg/mL of IL-1β, TNFα, IL-6, and IL-8 produced by macrophage-like cells after 4 hours **(A,C,E,G)** and 24 hours **(B,D,F,H)** of contact α-Ag2WO4 (7.81 µg/mL), β-Ag2MoO4 (15.62 µg/mL), and α-AgVO3 (3.9 µg/mL and 15.62 µg/mL) microcrystals. Error bars: standard deviation; CT: standard culture control; LPS: lipopolysaccharide, control. Groups with asterisks are statistically different from control. *: p < 0.0321; **: p = 0.0035; ***: p < 0.0039. α= .05.

For macrophage-like cells, all cytokines were detected (Figure 7). The production of TNFα, IL-6 and IL-1β was lower than CT after 4 h of contact with all silver-containing microcrystals (*p* ≤ 0.0321; Figures 7A, C, E). In contrast, when compared to control, no significant changes in the production of IL-8 were observed after 4 h of exposure to all experimental microcrystal (*p* ≥ 0.1789; Figure 7G). After 24 h of exposure to α-AgVO_3_ (15.62 μg/mL), macrophage-like cells showed a reduction in TNFα production (*p* = 0.0035) (Figure 7B). At this time, no significant changes in IL-6 production were observed, regardless the experimental microcrystals (*p* ≥ 0.1549; Figure 7D). Similar results were observed for IL-1β and IL-8, except for α-AgVO_3_ (15.62 μg/mL) and β-Ag_2_MoO_4_ (15.62 μg/mL), where there was an increased production of IL-1β (*p* = 0.0006) and IL-8 (*p* = 0.0039), respectively, after 24 h of exposure (Figures 7F, H).

### 3.5 Production of MMP-8 and -9

**FIGURE 8:**
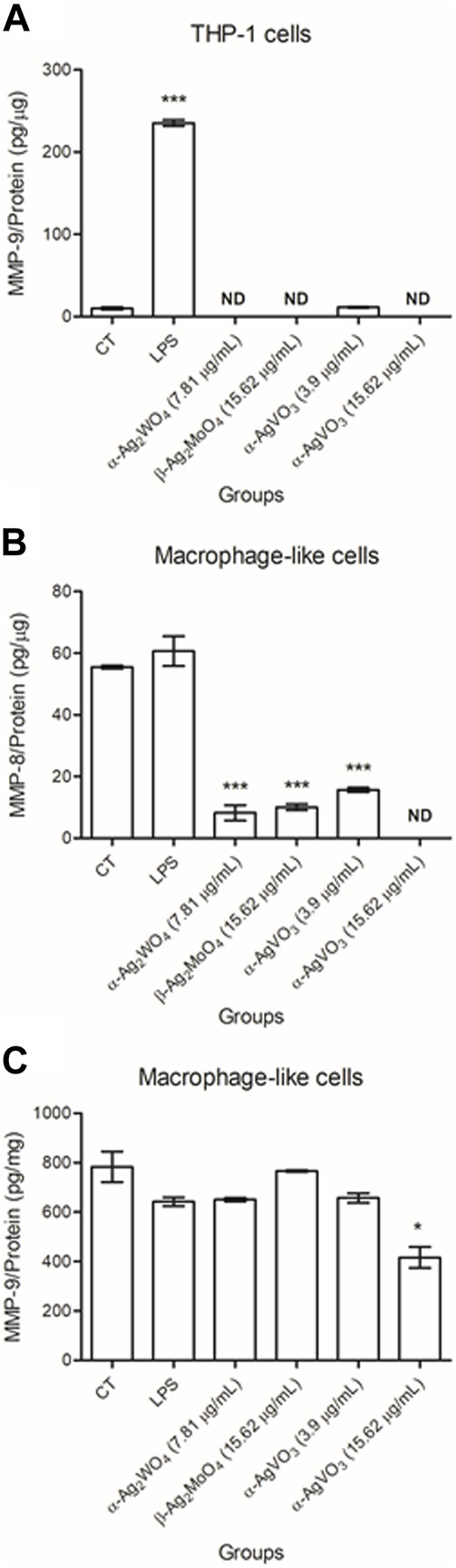
Mean values of pg/µg of MMP-9 released by THP-1 cells **(A)** and MMP-8 **(B)** and -9 **(C)** released by macrophage-like cells after 24 hours of contact with α-Ag2WO_4_ (7.81 µg/mL), β-Ag2MoO_4_ (15.62 µg/mL), and α-AgVO_3_ (3.9 µg/mL and 15.62 µg/mL) microcrystals. CT: standard culture conditions; LPS: lipopolysaccharide, positive control; ND: non-detected. Groups with asterisks are statistically different from control. *: *p* = 0.010; ***: *p* < 0.0001. α= .05.

The release of MMPs by THP-1 and macrophage-like cells, after 24 h of exposure to silver-containing microcrystals, was measured by the ELISA. It was not possible to detect the production of MMP-8 by THP-1 cells, even under standard cell culture conditions or in the presence of LPS (data not shown). The release of MMP-9 was not detected when these cells were stimulated with α-Ag_2_WO_4_ (7.81 μg/mL), β-Ag_2_MoO_4_ (15.62 μg/mL), and α-AgVO_3_ (15.62 μg/mL) (Figure 8A). The small amount of MMP-9 released after 24 h of contact with α-AgVO_3_ (3.9 μg/mL) was not statistically different from the control group (*p* = 0.7).

When evaluating the release of MMP-8 and -9 by macrophage-like cells, it was noted that all microcrystals promoted a decrease in the amount of MMP-8 released (*p* < 0.0001). Also, MMP-8 was not detected when these cells were maintained in contact with α-AgVO_3_ (15.62 μg/mL) for 24 h (Figure 8B). The exposure of macrophage-like cells to α-Ag_2_WO_4_ (7.81 μg/mL), α-AgVO_3_ (3.9 μg/mL), and α-AgVO_3_ (15.62 μg/mL) resulted in MMP-9 release statistically similar to control group (*p* ≥ 0.216; Figure 8C). Only macrophage-like cells exposed to β-Ag_2_MoO_4_ (15.62 μg/mL) for 24 h showed a decrease in MMP-9 released (*p* = 0.010).

**FIGURE 9:**
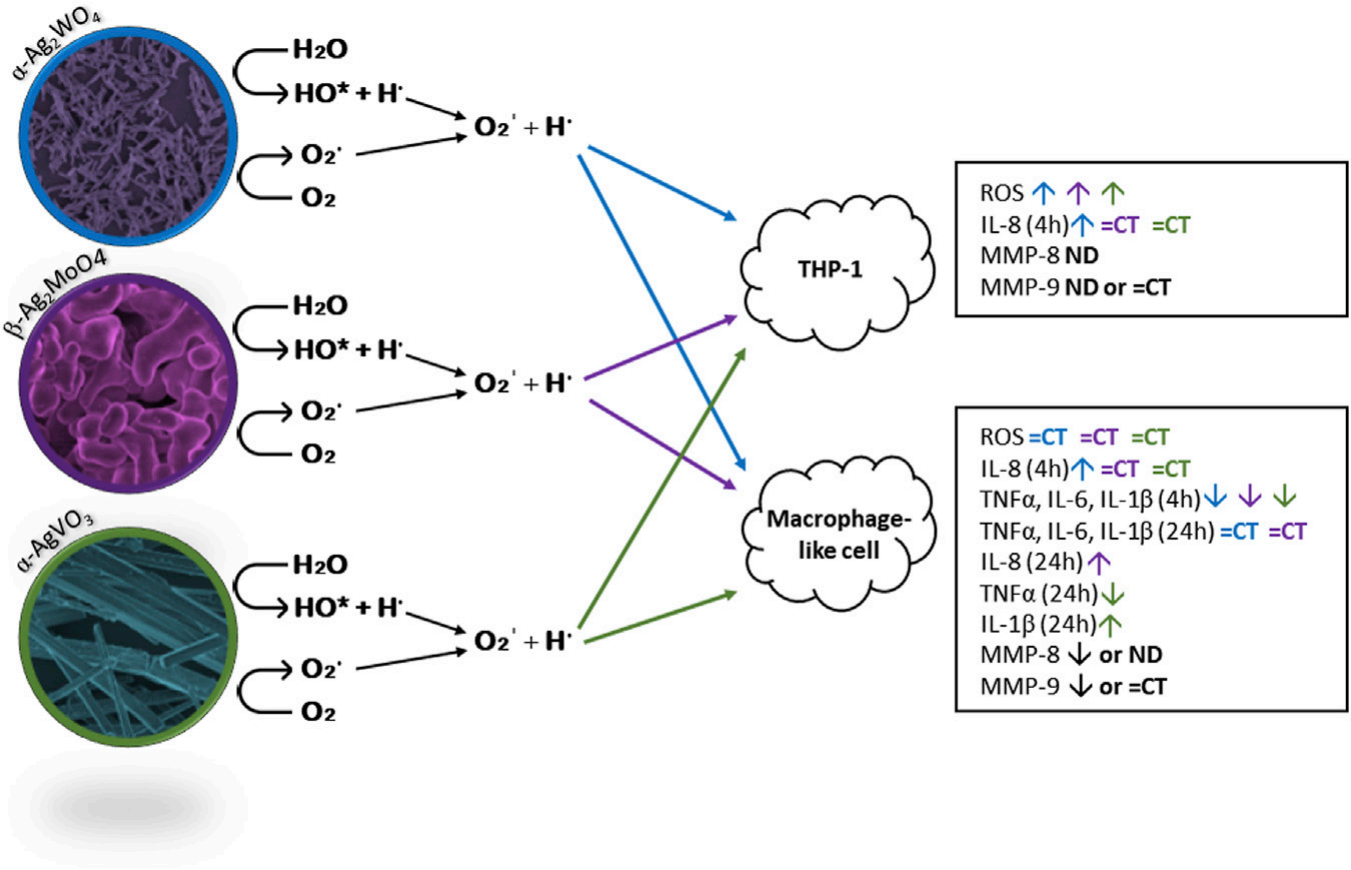
Schematic representation of ROS formation and its action on THP-1 and macrophage like cells, stimulating or inhibiting the production of ROS, pro-inflammatory cytokines, and MMPs as evaluated in this study. ND: non-detected; CT: control. α-Ag2WO_4_: blue arrows; β-Ag2MoO_4_: purple arrows; α-AgVO_3_: green arrows.

In Figure 9 we have a summary of the main findings of this work.

## 4 Discussion

The use of silver as an antimicrobial agent has been extensively studied in recent years. At the nanoscale, silver has demonstrated excellent antimicrobial properties by inducing the production of reactive oxygen species (ROS) (Carlson et al., 2008; Liu et al., 2010; Park et al., 2011; Akter et al., 2018; Canaparo et al., 2021; Liu et al., 2021). However, the generation of ROS can also be responsible for cytotoxic effects on mammalian cells (Carlson et al., 2008; Foldbjerg et al., 2009; Foldbjerg et al., 2011; Nishanth et al., 2011; Canaparo et al., 2021). Furthermore, silver concentration has been reported as a toxic factor within the range of 10–100 μg/mL (Chernousova and Epple, 2013). To enhance the antimicrobial properties of silver and improve its biocompatibility, researchers have combined silver with different metal oxides, such as vanadate (VO_3_), tungstate (WO_4_), and molybdate (MoO_4_). The compounds α-AgVO_3_, α-Ag_2_WO_4_, and β-Ag_2_MoO_4_ have shown antimicrobial activity against *C. albicans* (Fabbro et al., 2016; Foggi et al., 2017a; Foggi et al., 2017b; Assis et al., 2019; Pimentel et al., 2020; Pimentel et al., 2022), methicillin-resistant *S. aureus* (MRSA) (Longo et al., 2014; Oliveira et al., 2017; Assis et al., 2018; Foggi et al., 2020) and *E. coli* (Canaparo et al., 2021). They have been effective in reducing 3 log_10_ (CFU/mL) to 6 log_10_ (CFU/mL) (Longo et al., 2014; Fabbro et al., 2016; Foggi et al., 2017a; Foggi et al., 2017b; Oliveira et al., 2017; Pimentel et al., 2020; Pimentel et al., 2022), without causing damage to both human keratinocytes and fibroblasts cells (Haro Chávez et al., 2018; Assis et al., 2019; Pimentel et al., 2020). The results reported here indicate a slight decrease in cell viability after 24 h of contact with α- α-Ag_2_WO_4_ (7.81 μg/mL), β-Ag_2_MoO_4_ (15.62 μg/mL), and AgVO_3_ (3.9 μg/mL and 15.62 μg/mL) microcrystals. This decrease in viability may be partly attributed to the silver content and its ability to generate reactive oxygen species. Previous theoretical studies have suggested that these silver-containing microcrystals are formed by complex clusters connected by weak interactions and, when in an aqueous environment, these clusters can break water molecules into hydroxyl radicals and protons (OH* and H^•^). Simultaneously, there is an electron transfer to oxygen molecules (O_2_), resulting in the formation of Oʹ_2_, which interacts with the proton (H^•^) to form the radical HO_2_* (Fabbro et al., 2016; Foggi et al., 2017a; Oliveira et al., 2017).

The calculated silver content in the microcrystals used in this study was approximately 0.0156 μg/mL for α-Ag_2_WO_4_ (at 7.81 μg/mL), 0.0313 μg/mL for β-Ag_2_MoO_4_ (at 15.62 μg/mL), 0.0039 μg/mL for α-AgVO_3_ (at 3.9 μg/mL), and 0.0156 μg/mL for α-AgVO_3_ (at 15.62 μg/mL). At these concentrations, the silver-containing microcrystals were considered either non-cytotoxic or slightly cytotoxic. Such concentrations are significantly lower when compared to those found in silver nanoparticles described in previous studies (Martínez-Gutierrez et al., 2012; Martinez-Gutierrez et al., 2013).

It is already known that metal particles can indirectly induce ROS production due to the presence of metal ions (Haro Chávez et al., 2018; Assis et al., 2019). This oxidative stress can be responsible for the cytotoxicity of metal particles, considering that an increase in ROS generation can lead to cell damage and even cell death (Hashimoto et al., 2016; Canaparo et al., 2021; Liu et al., 2021). In this work, silver-containing microcrystals induced the production of superoxide (O_2_^−^) by THP-1 and macrophage-like cells. Interestingly, the production of superoxide by THP-1 cells when incubated with the silver-containing microcrystals was higher than that of H_2_O_2_ control. This is probably due to rapidly degradation of H_2_O_2_, limiting superoxide production by the cells. In contrast, the silver-containing microcrystals may promote a more sustained superoxide production. This is because, based on their mechanism of action, when in an aqueous environment, these silver-containing microcrystals degrade into complex clusters that interact with water and oxygen molecules, leading to the decomposition of these molecules into ROS (Foggi et al., 2017a; Foggi et al., 2017b; Oliveira et al., 2017; Foggi et al., 2020). When the NAC ROS scavenger was added to the cells, together with the microcrystals, the O_2_^−^ signaling was reversed. This was already expected because NAC is a ROS scavenger. In a previous study, Brzicova et al. (2019) also observed an increase in superoxide production after THP-1 cells were maintained in contact with silver nanoparticles for 24 h. However, no significant differences were observed among concentrations and times of exposition (Brzicova et al., 2019).

According to the literature, ROS, including anion superoxide (O_2_^•^^-^), activate the NF-κB (nuclear factor kappa B) and MAPK (mitogen-activated protein kinase) pathways, which stimulates the expression of genes responsible for IL-1β, TNFα and IL-6 production (Ndengele et al., 2005; Martínez-Gutierrez et al., 2012; Murphy et al., 2016; Yu et al., 2020; Canaparo et al., 2021). This may occur by oxidative stress, which is induced when the antioxidant ability of the cells is overcome by ROS generation (Park et al., 2011; Yu et al., 2020; Canaparo et al., 2021). In the present study, despite the high production of O_2_^−^ by THP-1 cells, there was no detection of IL-1β, TNFα, and IL-6. Only IL-8 was detected, but it was not significantly different from the control group, except for THP-1 cells in contact with α-Ag_2_WO_4_ (7.81 μg/mL) for 4h, where an increase in IL-8 production was observed. Furthermore, the exposure of macrophages-like to silver-containing microcrystals resulted in a decreased or similar production of the IL-1β, TNFα, IL-6, and IL-8 pro-inflammatory cytokines after 4 h. This decrease or similar production was maintained after 24 h for all cytokines evaluated, except for the increased production of IL-1β and IL-8, when macrophage-like cells were exposed to α-AgVO_3_ (15.62 μg/mL) and β-Ag_2_MoO_4_ (15.62 μg/mL), respectively. Previous findings have reported macrophage inflammatory responses caused by silver nanoparticles (Martínez-Gutierrez et al., 2012; Murphy et al., 2016). This may be attributed to the higher amount of ROS generated by silver nanoparticles due to their relatively large surface area (Park et al., 2011) compared to microcrystals. Another explanation is that nanoparticles can penetrate cell membranes and form clusters inside cell cytoplasm, inducing the inflammatory process (Martínez-Gutierrez et al., 2012), which does not occur with microcrystals due to their larger size. The findings reported here showed that even with the high production of O_2_^−^, this was easily reversed in the presence of a ROS scavenger, indicating that O_2_^−^ production by these particles may be self-limited and, consequently, less capable of inducing significant inflammatory responses. Thus, the low cytotoxicity of α-Ag_2_WO_4_ (7.81 μg/mL) and α-AgVO_3_ (3.9 μg/mL) could be explained by the reversible REDOX signaling by O_2_^−^, which is considered an important property of both microcrystals. Additionally, literature reports suggest that, among the ROS produced by cells, the O_2_^−^ pathway is less harmful (Schieber and Chandel, 2014).

The present investigation also reveled that when THP-1 and macrophage-like cells were stimulated with silver-containing microcrystals, the production of MMP-8 and MMP-9 decreased. Considering that TNFα is a physiological inducer of MMP-9 (Heidinger et al., 2006), the reduced amount of TNFα produced in the presence of silver-containing microcrystals may explain the decrease in MMP-9 production by the cells. Previous studies have demonstrated that MMPs play a role in pathological and healing processes in the oral environment, particularly in relation to periodontal disease, leading to the loss of periodontal attachment and bone destruction (Franco et al., 2017; Al-Majid et al., 2018; Zhang et al., 2018). Among the 23 types of MMPs identified so far, upregulation of MMP-8 and MMP-9 has been associated with periodontitis and peri-implantitis (Araújo et al., 2011; Franco et al., 2017; Al-Majid et al., 2018; Checchi et al., 2020), and other studies have reported that these two MMPs are linked to disease progression and bone loss (Arakawa et al., 2012; Al-Majid et al., 2018). Elevated levels of MMP-8 and MMP-9 are found in periodontal tissues where the disease is established, potentially indicating the severity and progression of the pathology (Franco et al., 2017; Al-Majid et al., 2018; Checchi et al., 2020). Moreover, MMP-8 has been implicated in bone loss in patients with severe peri-implantitis (Arakawa et al., 2012; Al-Majid et al., 2018).

Hashimoto et al. (2016) evaluated cytotoxicity, genotoxicity, and MMP production of gold and platinum nanoparticles on human cells were evaluated, along with their effect on dental resin properties (Hashimoto et al., 2016). The authors demonstrated that gold nanoparticles inhibited MMP production without causing cell damage, which is an interesting characteristic considering that MMP production can contribute to the failure of dental restorations (Hashimoto et al., 2016). Therefore, therapies that can reduce the production of MMP-8 and MMP-9 may be effective in preventing peri-implant disease.

The favorable biological responses of the α-Ag_2_WO_4_, β-Ag_2_MoO_4_, and α-AgVO_3_ microcrystals in the present investigation, along with studies highlighting their antimicrobial properties, suggest that these microcrystals are promising candidates as coating materials for dental and medical devices.

## 5 Conclusion

In conclusion, α-Ag_2_WO_4_ (7.81 μg/mL), β-Ag_2_MoO_4_ (15.62 μg/mL), and α-AgVO_3_ (3.9 μg/mL and 15.62 μg/mL) demonstrated low cytotoxicity to THP-1 and macrophage-like cells over a sufficiently long period to measure potential damage. Additionally, these microcrystals increased the production of O_2_^−^ and modulated cytokines and MMP production in a cell phenotype-dependent manner. The data presented here indicated that α-Ag_2_WO_4_ (7.81 μg/mL), β-Ag_2_MoO_4_ (15.62 μg/mL), and α-AgVO_3_ (3.9 μg/mL and 15.62 μg/mL) are capable of modulating immune response by either increasing or decreasing the production of key pro-inflammatory cytokines. Thus, the potential future applications of these microcrystals in the dental and medical fields appear promising and warrant further evaluation.

## Data Availability

The original contributions presented in the study are included in the article/supplementary material, further inquiries can be directed to the corresponding author.
